# Radiolabeled Somatostatin Analogues for Diagnosis and Treatment of Neuroendocrine Tumors

**DOI:** 10.3390/cancers14041055

**Published:** 2022-02-19

**Authors:** Valentina Ambrosini, Lucia Zanoni, Angelina Filice, Giuseppe Lamberti, Giulia Argalia, Emilia Fortunati, Davide Campana, Annibale Versari, Stefano Fanti

**Affiliations:** 1 Department of Experimental Diagnostic and Specialized Medicine, University of Bologna, 40138 Bologna, Italy; valentina.ambrosini@unibo.it (V.A.); giuseppe.lamberti8@unibo.it (G.L.); giulia.argalia@studio.unibo.it (G.A.); emilia.fortunati@studio.unibo.it (E.F.); davide.campana@unibo.it (D.C.); stefano.fanti@aosp.bo.it (S.F.); 2 Nuclear Medicine Unit, IRCCS Azienda Ospedaliero-Universitaria di Bologna, 40138 Bologna, Italy; 3 Nuclear Medicine Unit, Azienda USL-IRCCS di Reggio Emilia, 42123 Reggio Emilia, Italy; angelina.filice@ausl.re.it (A.F.); annibale.versari@asmn.re.it (A.V.); 4 Division of Medical Oncology, IRCCS Azienda Ospedaliero-Universitaria di Bologna, 40138 Bologna, Italy

**Keywords:** somatostatin, neuroendocrine, [Ga]-DOTA peptides PET/TC, PRRT

## Abstract

**Simple Summary:**

Neuroendocrine neoplasms (NENs) are rare and heterogeneous tumors, presenting in often challenging clinical scenarios, and require multidisciplinary discussion for optimal care. The theranostic approach (DOTA peptides labelled with ^68^Ga for imaging well-differentiated neuroendocrine tumors NETs, and labelled with ^90^Y or ^177^Lu for therapy) plays a crucial role in the management of NENs to assess disease extension and criteria for peptide receptor radionuclide therapy (PRRT) eligibility of based on somatostatin receptor (SSTR) expression. The present paper is an overview of currently employed radiolabeled SSTR analogues used for both diagnosis and therapy of NENs. Further emerging radiopharmaceuticals targeting SSTRs (e.g., fluorinated SSTR agonists, radiolabeled SSTR antagonists) as well as strategies to improve PRRT efficacy (by means of implementation of personalized treatment schemes, dosimetry, amelioration of response assessment strategies, and optimization of treatment sequencing) are also discussed. Finally, although very preliminary, some studies employing radiomic features in various kinds of NET are reported.

**Abstract:**

Neuroendocrine neoplasms (NENs) are rare and heterogeneous tumors that require multidisciplinary discussion for optimal care. The theranostic approach (DOTA peptides labelled with ^68^Ga for diagnosis and with ^90^Y or ^177^Lu for therapy) plays a crucial role in the management of NENs to assess disease extension and as a criteria for peptide receptor radionuclide therapy (PRRT) eligibility based on somatostatin receptor (SSTR) expression. On the diagnostic side, [^68^Ga]Ga-DOTA peptides PET/CT (SSTR PET/CT) is the gold standard for imaging well-differentiated SSTR-expressing neuroendocrine tumors (NETs). [^18^F]FDG PET/CT is useful in higher grade NENs (NET G2 with Ki-67 > 10% and NET G3; NEC) for more accurate disease characterization and prognostication. Promising emerging radiopharmaceuticals include somatostatin analogues labelled with ^18^F (to overcome the limits imposed by ^68^Ga), and SSTR antagonists (for both diagnosis and therapy). On the therapeutic side, the evidence gathered over the past two decades indicates that PRRT is to be considered as an effective and safe treatment option for SSTR-expressing NETs, and is currently included in the therapeutic algorithms of the main scientific societies. The positioning of PRRT in the treatment sequence, as well as treatment personalization (e.g., tailored dosimetry, re-treatment, selection criteria, and combination with other alternative treatment options), is warranted in order to improve its efficacy while reducing toxicity. Although very preliminary (being mostly hampered by lack of methodological standardization, especially regarding feature selection/extraction) and often including small patient cohorts, radiomic studies in NETs are also presented. To date, the implementation of radiomics in clinical practice is still unclear. The purpose of this review is to offer an overview of radiolabeled SSTR analogues for theranostic use in NENs.

## 1. Introduction

Neuroendocrine neoplasms (NENs) account for 0.5% of all malignancies; they are rare, heterogeneous (in terms of primary tumor site, behavior over time, and differentiation grade), and mostly slow-growing and non-functioning, originating from the secretory cells of the neuroendocrine system, and widely dispersed in the human body. The majority of NENs (72%) arise from the gastroenteropancreatic (GEP) tract, followed by the bronchopulmonary system (25%), while other primary sites are less frequent (e.g., adrenals, thyroid, breast, prostate, skin). A minority of cases present as inherited syndromes (e.g., von Hippel–Lindau disease (VHL), multiple endocrine neoplasia (MEN), neurofibromatosis, tuberous sclerosis). NENs are pathologically classified depending on cells’ morphology and differentiation (Ki-67 and the mitotic count) into well-differentiated tumors (NETs) (graded as NET G1: Ki-67 < 3% or mitotic count per 10 high-power fields < 2; NET G2: Ki-67 with 3–20% or 2–20 mitoses; NET G3 Ki-67 > 20% or > 20 mitoses) [1,2] and poorly differentiated neuroendocrine carcinoma (NEC, Ki-67 > 20%). 

The diagnostic workup of NENs is challenging, mostly because they often present as small lesions with variable anatomical localization. 

Conventional imaging (CI) procedures (e.g., ultrasound (US), diagnostic computed tomography (dCT), magnetic resonance imaging (MRI)) are routinely employed [3,4,5] for imaging of NENs; the sensitivity and detection rate of dCT are suboptimal (NET disease: sensitivity range (61–93%) and specificity range (71–100%); liver metastases: sensitivity range (75–100%) and specificity (83–100%); nodes: sensitivity range (60–70%) and specificity (87–100%)) [6,7,8]. For imaging of the abdomen, bone, and brain, MRI is generally better than dCT. Endoscopic US is the most sensitive method for diagnosing pancreatic NETs (mean sensitivity = 86% (82–93%); mean specificity = 92% (86–95%)), and CEUS may be considered for localization of NET liver metastases and pancreatic NETs [6,8]. 

The diagnostic workup of NENs was therefore revolutionized by the introduction of nuclear medical procedures able to detect the presence of somatostatin receptors (SSTRs), which are typically overexpressed in well-differentiated NEN cells. 

Over the years, several studies have indicated that SSTR PET/CT (positron emission tomography) imaging is superior to SSTR scintigraphy or conventional anatomic imaging (US, dCT, or MRI) for the assessment of well-differentiated NETs. For example, SSTR PET/CT can locate the primary tumor site, and often demonstrates additional lesions not captured by conventional imaging, resulting in more accurate evaluation of disease extension, followed by relevant changes in management in approximately one-third of patients [9,10].

Furthermore, SSTR agonists (e.g., DOTA-TOC, DOTA-NOC, DOTA-TATE) currently used for PET/CT imaging allow not only the evaluation of disease extension when labelled with ^68^Ga, but also the selection of candidate patients for target therapy with either non-radioactive or radioactive ([^90^Y]Y-DOTA-TOC or [^177^Lu]Lu-DOTATATE) somatostatin analogues. 

The present paper discusses currently clinically employed radiopharmaceuticals—agonists of the SSTR—for both diagnosis and therapy of NENs.

## 2. Imaging of NENs with Radiolabeled Somatostatin Analogues

SSTR expression can be demonstrated in vivo by radiolabeled somatostatin analogues using planar scintigraphy, single-photon-emission computed tomography (SPECT)/CT, or PET/CT, the latter being the imaging modality of choice when available. 

In fact, the first agent to obtain Food and Drug Administration (FDA) approval in 1994 for planar and SPECT imaging was [^111^In]In-pentetreotide (Octreoscan^TM^); it was, however, characterized by less favorable dosimetry and lower diagnostic performance (detection rate ranging between 50% and 100% [6,7,8]) when compared with [^68^Ga]-DOTA peptides ([^68^Ga]Ga-DOTA-TOC, [^68^Ga]Ga-DOTA-NOC, [^68^Ga]Ga-DOTA-TATE), mostly due to the suboptimal physical characteristics of the radiopharmaceutical, high physiological liver uptake (frequent site of NET metastases), gamma camera’s lower spatial resolution, and increased patient discomfort due to later/longer acquisition times for imaging [9].

Since its first introduction in clinical practice, SSTR PET/CT has demonstrated several advantages over scintigraphic imaging (i.e., higher spatial resolution, more favorable biodistribution at liver/bowel level, semi-quantitative analysis), and currently represents the gold standard functional imaging modality for NETs, and is recommended by all guidelines [6,8]. 

The radiopharmaceuticals currently used for SSTR PET/CT present a common structure: a positron-emitting isotope (^68^Gallium, ^68^Ga), a chelant (DOTA), and the SSTR ligand (NOC, TOC, TATE). This corresponds to three clinically employed tracers—[^68^Ga]Ga-DOTA-TOC, [^68^Ga]Ga-DOTA-NOC, and [^68^Ga]Ga-DOTA-TATE—showing variable affinity for the SSTR subtypes.

^68^Ga is a positron-emitting radionuclide that can only be applied for diagnostic imaging; its short half-life (68 min) makes it unsuitable for dosimetry, and therefore inapplicable for therapeutic purposes. Different types of ^68^Ge/^68^Ga generators are currently available with marketing authorization and using kit-based radiolabeling. The radiopeptides function as receptor agonists to SSTR through the activation and internalization of the receptor upon binding. Despite different SSTR subtypes’ affinities, the clinical performance of the available radiopharmaceuticals is considered clinically comparable, since they all bind with high affinity to SSTR-2—the subtype predominantly expressed in most NENs.

[^68^Ga]Ga-DOTA-TOC was the first PET SSTR ligand, developed in 2001 and approved in Europe in 2016, and by the FDA in 2019 [11]; it has an octreotide-like SSTR affinity profile—indeed, it has affinity for SSTR-2 and -5, but lower compared to the [^68^Ga]Ga-DOTA-NOC. SSTR-2 expression in immunohistochemistry demonstrated a significant correlation with the standardized uptake value (SUV) [12]. DOTATOC can also be labelled with ^90^Y for theranostic applications.

[^68^Ga]Ga-DOTA-lanreotide ([68Ga]Ga-DOTA-LAN) binds to SSTR-2 and -5 [13].

[^68^Ga]Ga-DOTA-NOC has high affinity for SSTR-2,3,5 [14].

[^68^Ga]Ga-DOTA-TATE, which received FDA approval in 2016 (https://www.accessdata.fda.gov/drugsatfda_docs/label/2018/208547s011lbl.pdf accessed on 6 November 2021), shows the highest affinity for SSTR-2, with a higher tumor-to-background ratio (TBR) compared to [^68^Ga]Ga-DOTA-NOC [15]; moreover, when labelled with ^177^Lu, it can be employed for NET peptide receptor radionuclide therapy (PRRT), approved by the EMA in 2017 and by the FDA in 2018 (https://www.accessdata.fda.gov/drugsatfda_docs/label/2018/208700s000lbl.pdf accessed on 6 November 2021). For diagnostic purposes, DOTATATE was also labelled with ^64^Cu [16]; such labelling offers a longer radionuclide half-life (12.7 h of ^64^Cu vs. 68min of ^68^Ga), shorter positron range in tissue (mean 0.6 vs. 3.5 mm), and lower positron branching ratio (0.17 vs. 0.89), resulting in higher TBR at delayed images (acquired 3–24 h p.i.). [^64^Cu]Cu-DOTA-TATE received FDA approval in 2020 (https://www.accessdata.fda.gov/drugsatfda_docs/label/2020/213227s000lbl.pdf accessed on 6 November 2021).

### 2.1. SSTR PET/CT Indications

The procedural guidelines of the European Association of Nuclear Medicine (EANM) for the use of PET/CT imaging with [^68^Ga]Ga-DOTA-peptides were last updated in 2017 [14].

According to the 2017 EANM guidelines, SSTR PET/CT is recommended as the first choice to study patients with foregut and midgut NETs and head and neck paraganglioma; it is generally considered to be the second choice for abdominal paraganglioma (second to [^18^F]F-DOPA) and hindgut NETs (second to [^18^F]FDG). Finally, SSTR PET/CT is indicated as the third choice for medullary thyroid carcinoma (MTC) [14].

EANM guidelines recommend SSTR PET/CT for:

- Detection of primary occult site (CUP) in patients with demonstrated neuroendocrine metastasis or with increased specific tumor markers, with negative conventional imaging for primary lesions;
- Characterization of bronchial masses with inconclusive conventional imaging;
- Characterization, staging, and restaging of foregut NETs;
- Characterization, staging, and restaging of midgut NETs when [^18^F]F-DOPA was unavailable or inconclusive.

Regarding patient management, SSTR-PET/CT is indicated for:

- Staging/restaging of residual or recurrent disease;
- Prognosis (since SSTR-positive tumors are more likely to respond to targeted somatostatin analogue therapy);
- Assessment of eligibility for PRRT (with [^177^Lu]Lu or [^90^Y]Y-DOTA-peptides);
- Therapy response monitoring (surgery, radiotherapy, chemotherapy, or PRRT).

Although a large body of literature is available on NENs, many systematic reviews and meta-analyses suggest that strong clinical evidence is often difficult to achieve, mostly due to the rarity, clinical heterogeneity, and lack of methodological standardization of imaging and clinical studies. In fact, the clinical management and diagnostic workup often varies between countries, influenced by both instrumental availability and reimbursement policies.

A step towards standardization was made in 2008, when the ENETS (European Neuroendocrine Tumor Society) set up an accreditation/certification program that allows participating centers to display the expertise of their multidisciplinary teams’ knowledge of and adherence to ENETS guidelines for patient care, in order to increase patient-oriented care and participation in clinical trials, as well as collaboration between centers. Since 2009, 62 ENETS Centers of Excellence (CoEs) have been accredited worldwide [15].

In order to further define the appropriate use criteria (AUC, RAND/UCLA Appropriateness Method) for SSTR PET/CT, a pool of expert representatives of several societies (the Society of Nuclear Medicine and Molecular Imaging, the American College of Radiology, the American Society of Clinical Oncology, the North American Neuroendocrine Tumor Society, the European Association of Nuclear Medicine, the Endocrine Society, the Society of Surgical Oncology, the National Comprehensive Cancer Network, the American College of Physicians, the American Gastroenterological Association, and the World Conference on Interventional Oncology) gathered to rate 12 clinical scenarios [17]; 9 out of 12 were considered appropriate: initial staging after the histological diagnosis of NET; localization of a primary tumor in patients with known metastatic disease but an unknown primary; selection of patients for PRRT; staging of NETs prior to planned surgery; evaluation of a mass suggestive of an NET not amenable to endoscopic or percutaneous biopsy (e.g., ileal lesion, hypervascular pancreatic mass, mesenteric mass); monitoring of NETs seen predominantly on SSTR-PET; evaluation of patients with biochemical evidence and symptoms of an NET without evidence of it on CI, and without prior histological diagnosis of an NET; restaging at the time of clinical or laboratory progression without progression on CI; and new indeterminate lesion on CI with unclear progression.

More recently, a harmonization project was promoted by the EANM Focus 3 Consensus [18,19]; a multidisciplinary modified Delphi process (including 24 international experts) was applied to assess the most debated issues in the management of well-differentiated NETs, in order to reach a final consensus. SSTR PET/CT was recommended for unknown primary NETs, metastatic NETs, NET staging/restaging, suspected extra-adrenal pheochromocytoma/paraganglioma, and suspected paraganglioma.

### 2.2. SSTR PET/CT Procedure

SSTR PET/CT procedural guidelines were published by the EANM and ENETS in 2017 [6,8,14]. The recommended dose is between 100 and 200 MBq. Uptake time is slightly different between conjugated peptides: 45–60 min for TATE, and 60–90 min for TOC and NOC; there is no need for fasting.

A temporary withdrawal before PET examination of medications with “cold” somatostatin analogues is preferable (to avoid SSTR blockade), but not strictly necessary. In case of discontinuation, 1 day is recommended in the case of short-acting molecules, whereas 3–4 weeks is recommended for long-acting molecules. The optimal timing would be to perform the scan just before the scheduled monthly dose of long-acting analogues [14].

Precautions should be taken in case of pregnancy and subjects < 18 years (benefit vs. risk evaluation), as well as breastfeeding (interruption for 7 physical half-lives of the administered radionuclide).

### 2.3. [^68^Ga]Ga-DOTA Peptides’ Biodistribution and Pitfalls

The maximum tumor activity was found at 70 ± 20 min post-injection (pi) [20]. Excretion was predominately via kidney. Physiological uptake was demonstrated in the liver, spleen (by far the site of highest uptake), pituitary, thyroid, and salivary glands (faint), kidneys, adrenal glands, stomach, bowel (linear, non-focal), prostate gland, and breast. Variable uptake was found in the pancreas; in particular, the expression of SSTR-2 was detected in the islets—especially in the pancreatic head. The pattern of uptake of the uncinate process/head of the pancreas can be focal or diffuse, and may present as higher than that of the liver. For definitive image interpretation, a negative dCT confirming the absence of a morphologically evident lesion is mandatory.

Although the liver is a frequent site of NENs’ metastatic spread, the detection of lesions presenting an uptake higher than the liver is generally a positivity criterion for pathological SSTR expression.

Various false-negative findings were encountered, especially lesion de-differentiation, histotypes with absent or variable SSTR expression (e.g., medullary thyroid carcinoma, neuroblastoma, insulinoma, pheochromocytoma), small lesions (under PET/CT spatial resolution), and lesions at sites of physiological biodistribution (hampering tumor detectability).

False-positive findings were also frequent; SSTR increased expression of activated lymphocytes at sites of inflammation/infection, non-neuroendocrine tumors expressing SSTR (including breast, prostate, head and neck, renal cell, differentiated thyroid carcinoma, non-small-cell lung cancer, melanoma, lymphoma, sarcoma, and astrocytoma), accessory spleens (including intrapancreatic ones), and splenules [14].

### 2.4. SSTR PET/CT Diagnostic Performance and Clinical Impact

The data collected in the past decade certainly prove that nuclear medicine procedures have the highest accuracy for the detection of NET lesions, and strongly influence the patients’ clinical management.

As previously mentioned, several studies have reported the higher diagnostic accuracy of SSTR PET/CT as compared to somatostatin receptor scintigraphy and CT [9,21,22].

Moreover, published data indicate that the results obtained with the currently available SSTR agonists are clinically equivalent (although semi-quantitative parameters’ absolute values may differ when using different compounds, rendering absolute SUV values not directly comparable).

A large cohort study (728 patients with confirmed NET/suspected NET and different primary tumor sites; 1258 PET/CT scans) performed at a single institution reported that the diagnostic performance of SSTR PET/CT ([^68^Ga]Ga-DOTA-TATE) was very high regardless of the primary tumor site or of the indications for PET/CT scanning (in particular, sensitivity, specificity, accuracy, positive predictive value, and negative predictive value were 97%, 95.1%, 96.6%, 98.5%, and 90.4%, respectively) [23].

The superiority of SSTR PET/CT as compared to CT was reported in particular for the detection of small lesions, as well as nodal and bone metastases [9,24,25]. A dramatic decrease in survival was in fact reported for patients with bone involvement on [^68^Ga]Ga-DOTA-TATE PET/CT; patients without signs of metastasis on [^68^Ga]Ga-DOTA-TATE PET/CT showed a longer overall survival (of 95.7%, 88.6%, and 81.3% at 1, 3, and 5 y, respectively) compared with patients with soft-tissue metastasis (90.5%, 82.3%, and 71.7%, respectively) or with bone metastasis (85.6%, 60.5%, and 44.1%, respectively) [23].

Interobserver agreement in SSTR PET/CT image interpretation was almost perfect when 7 nuclear medicine physicians from 5 different institutions were asked to review SSTR PET/CT scans of 50 NET patients. In particular, reproducibility ranged from substantial to almost perfect for overall scan results and organ/lymph node involvement, and an almost perfect interobserver agreement was also observed for tumor SUVmax assessment (intraclass correlation coefficient 0.99). Liver SUVmax and spleen SUVmean were also highly reproducible (intraclass correlation coefficients of 0.79 and 0.81, respectively) [26].

Considering the strong link between SSTR expression and receptor-targeted treatment options, SSTR PET/CT strongly influences patients’ management. SSTR PET/CT-derived information has been reported to strongly impact the clinical management (from 30% to 50% of the cases), by changing either the stage or the clinical strategy [10,23,27].

In a cohort of 90 pathologically confirmed NET cases, DOTANOC PET/CT impacted management in slightly more than half of the patients (50/90 cases, 55%). Most patients initiated/continued PRRT (27 patients) or SSA (7 patients) treatment after SSTR PET/CT, or were referred to surgery (6 patients). Less common conditions were exclusion from previously planned surgery or PRRT (in SSTR-negative cases), indication to liver transplantation or radiotherapy, and further diagnostic workup [27].

Similar results were portrayed in a much larger study including 1258 PET/CT scans; the treatment plan was changed after 515/1258 (40.9%) [^68^Ga]Ga-DOTA-TATE PET/CT scans because of new, unexpected findings. In most cases, the new treatment comprised initiation of chemotherapy or PRRT (362/515, 70.3%). In 10.1% (52/515), PET/CT-derived data were followed by surgery, while in 13.8% (71/515) a new chemotherapy regimen was administered. Less common treatment changes included cessation of previous treatment, exclusion from PRRT, and indication for liver transplant [23].

In an effort to collect stronger clinical evidence, overcoming the limits imposed by the often small numbers of patients included in published cohorts (mostly due to the rarity of the disorder), meta-analytic data were extrapolated. In particular, a recently published umbrella review collected the results of 34 meta-analyses [28], analyzing the diagnostic performance of different radiopharmaceuticals (SSTR PET/CT, [^18^F]F-DOPA PET/CT, [^18^F]FFDG PET/CT) in NENs or suspected NETs. The main results derived from meta-analyses of the diagnostic performance of SSTR PET/CT are reported in Table 1 [29,30,31,32,33,34,35,36,37,38,39,40,41,42].

Overall, SSTR PET/CT (using either NOC, TOC, or TATE) is the standard and preferred imaging modality to functionally study well-differentiated NETs, showing high per-patient pooled sensitivity and specificity respectively (both > 90% for lung and GEP NETs). SSTR PET/CT is also valuable for the detection of head and neck paraganglioma, while it shows a lower detection rate for abdominal forms. Lower accuracy is generally expected for MTC and CUP 1.

Meta-analysis data also confirm that a change in management occurs in more than one-third of the cases. In particular a systematic review and meta-analysis evaluated the clinical impact of SSTR PET/CT, analyzing a total of 1561 NET patients in 14 studies. Overall, a change in management occurred in 44% (range, 16–71%) of patients. Furthermore 7 of 14 studies differentiated between inter- and intramodality changes, with most changes being intermodality (77% vs. 23%) [10].

### 2.5. Quantification

Quantification of radiopharmaceuticals’ uptake is recommended in PET/CT reports, and may provide an indirect measure of cells’ differentiation. In fact, SSTR PET/CT sensitivity varies between tumor types on the basis of SSTR density; high uptake has been largely demonstrated in most low-grade, highly differentiated NETs (i.e., GEP, lung NETs, sympathoadrenal system tumors, meningioma), usually expressing high SSTR density.

In 2010, Campana et al. reported that [^68^Ga]Ga-DOTA-NOC PET/CT SUVmax was correlated with the clinical and pathological features of NETs, and might therefore be used for prognostication (in fact, lesions with higher SUVmax and, therefore, higher SSTR expression, have a lower grade and a better prognosis). In line with these data, similar results were observed in pancreatic G1 and G2 NETs; significant differences in progression-free survival (PFS) were observed in relation to Ki-67 (median, 45 months for Ki-67 ≤ 5% and 20 months for Ki-67 > 5%; *p* = 0.005), SUVmax (<37.8 vs. >38.0: 16.0 vs. 27.0 months; *p* = 0.002), and type of therapy (medical vs. peptide receptor radionuclide therapy: 16.0 vs. 26.0 months; *p* = 0.014) [43].

Interestingly, in 10 patients with metastatic neuroendocrine tumors who underwent a 45-minute dynamic acquisition followed by three whole-body PET/CT examinations at 1, 2, and 3 h after injection with both [^68^Ga]Ga-DOTA-TOC and [^68^Ga]Ga-DOTA-TATE, SUV did not correlate linearly with Ki-67, and achieved saturation for a Ki of greater than 0.2 mL/cm^3^/min, corresponding to an SUV of more than 25. Thus, SUV may not reflect the SSTR density accurately at its higher values. Therefore, in patients with elevated SSTR expression and a large tumor burden, a limiting step for tracer uptake might be due to plasma peptide availability and receptor saturation [44].

In fact, from a merely prognostic point of view, the radiopharmaceutical that is most certainly able to provide more clinically relevant prognostic data is [^18^F]FDG, since it can demonstrate the presence of more aggressive clones that will ultimately drive the patient’s prognosis.

SUVmax thresholds were also proposed to select patients eligible for PRRT. In a study including 40 patients with advanced-stage NETs treated with a fixed dose of [^90^Y]Y-DOTA-TOC (5550 or 3700 MBq), using a [^68^Ga]Ga-DOTA-TOC SUVmax > 17.9 as the cutoff for favorable outcomes, PET was able to predict the treatment response of all responders and of 15/16 non-responders. All four patients with equivocal findings showed an SUV ≤ 17.9, and soon experienced tumor progression [45].

A comparable threshold was found by a group from Heidelberg, assessing 60 liver metastases in 30 NET patients at baseline and after PRRT. Using a threshold value of >16.4 for SUVmax, the sensitivity and specificity in predicting responding lesions were 95 and 60%, respectively [46].

However, it is well known that absolute SUV values are affected by several biases, especially due to the difficulty of standardization between different scanners and technologies. The tumor-to-liver ratio (TLR, calculated as lesion SUVmax divided by SUVmean of the liver) and tumor-to-spleen ratio (TSR) are scanner-independent criteria that enable the translation of results to other institutions. In the same German study, [^68^Ga]Ga-DOTA-TOC TLR of 2.2 was predictive of a good response [46].

In a recent meta-analysis, a high interobserver agreement on the overall scan interpretation as well as in the assessment of SUVmax values (of the primary tumor as well as in the liver background) was reported [26].

Volumetric functional features have been also explored; for example, SSTR-expressing tumor volume (SRETV) (e.g., segmented at 50% of the SUVmax) and total lesion SSTR (TLSRE = SSTR-expressing tumor volume × tumor SUVmean). In particular, WHO tumor grade and the sum of all SRETVs have been reported to be independent predictors of PFS [47].

Certainly, SUVmax values should always be interpreted critically, taking into account the many factors that can affect its measure; however, it is important to underline that SUVmax can provide valuable data, especially regarding whole tumor volume, cells’ differentiation, and the potential heterogeneity of SSTR expression, all of which will ultimately have an impact on management. Therefore, it is clinically relevant to indicate the SUVmax value of the most relevant lesions (e.g., the ones with the highest uptake, or lesions presenting a low SUVmax that may require further investigation).

From a clinical point of view, all lesions presenting an uptake higher than that of the liver are considered as presenting pathological SSTR expression. This criterion may, however, not be applicable to very small lesions (affected by partial volume effect, e.g., millimetric ileum NET) or in cases with extensive metastatic liver involvement (in which the assessment of SUVmax of the liver background is practically impossible).

The visual assessment of uptake intensity by comparing tumor uptake with the liver background was first proposed by means of the Krenning score—a visual five-point scale first developed for Octreoscan^TM^ in 1993 [48]. More recently, the scale was adapted to fit SSTR PET/CT as the modified Krenning score [49], and is currently applied in clinical practice to guide PRRT eligibility (0: no uptake, <blood pool; 1: very low, <liver; 2: = liver; 3: >liver but <spleen; 4: = or >spleen). Head-to-head analysis demonstrates higher Krenning scores for [^68^Ga]Ga-DOTA-TATE PET/CT compared to ^111^In-pentetreotide, particularly when lesions measure 2 cm or less. In fact, small lesion size resulted in low Krenning scores using ^111^In-pentetreotide, but lesion size did not affect SSTR PET-based Krenning scores [49]. The use of a visual criterion of uptake (uptake equal or higher than the liver) is also recommended by the American College of Radiology (ACR), for [^68^Ga]Ga-DOTA-TATE [50] to identify patients likely to respond SSTR targeted treatments.

### 2.6. Standardized Reporting

A structured reporting system, entitled SSTR-RADS version 1.0, was proposed in the effort to standardize assessment for both diagnosis and treatment planning in neuroendocrine tumors. This system consists of a five-point scale assessing the likelihood of malignancy (from grade 1—negligible, benign—to grade 5: high probability of NET), a three-point score of the uptake intensity of the largest and most avid lesion, and a summed RAD score considering the scores of five target lesions [51,52,53].

In line with the concept of medical practice harmonization, a novel ENETS standardized (synoptic) reporting system was proposed [54]; a multidisciplinary panel performed a wide discussion and literature review in order to develop a synoptic template for SSTR PET/CT reporting. Nuclear medicine readers are encouraged to implement the following elements in their current reporting practice:

- Clinical details (e.g., indication, primary location, pathology, inherited/clinical syndrome);
- Procedure (e.g., radiopharmaceutical, administered activity, uptake time, scan type, field of view, interventions);
- Comparative imaging (e.g., modality, radiopharmaceutical);
- Findings (e.g., primary location: size and avidity using Krenning score; nodes: locoregional vs. distant; metastases: locations and characteristics, e.g., number, size, heterogeneity, necrosis, Krenning score);
- For reference lesions, location, visual avidity (e.g., Krenning score), semi-quantitative avidity (e.g., SUVmax, TSR, SSTR–FDG relationship), and size (uni- or bidimensional) should be included [54].

### 2.7. SSTR for Response Assessment

The role of SSTR PET/CT is still unclear for the response assessment scenario; EANM 2017 guidelines recommend the use of RECIST for response assessment [14]. ENETS 2017 guidelines also recommend the use of the RECIST criteria in PanNETs [6].

Although the RECIST criteria represent the gold standard to assess response in oncology, they are not ideal in the NEN setting, since these are typically slow-growing tumors that may present cystic components. The integration of morphological features with density data (CHOI criteria, originally applied to gastrointestinal stromal tumors) was proposed but not validated [55].

To date, no definite response criteria have been validated for PET/CT. Moreover, PET/CT is not routinely performed at interim, nor at the end of treatment. It is well known that variations in the lesions’ SUVmax values are not always an indicator of response (e.g., reduced uptake after therapy might reflect a good response but also a loss of SSTR expression due to tumor dedifferentiation [17]. SUVmax alone does not account for intralesional variation of SSTR expression, and further prospective studies are warranted to validate the other quantitative parameters already reported in the previous paragraph (see Section 2.5). In clinical practice, it is crucial to identify the disappearance of previous lesions or the appearance of new lesions rather than to measure SUV changes.

Even at the recent EANM FOCUS3 consensus, there was no agreement on the best imaging modality for PRRT treatment monitoring. However, it is interesting to note that the majority of panelists (approximately 58%) voted for a combination of SSTR PET/CT and triple-phase contrast-enhanced dCT and/or MRI as their preferred imaging modality [18,19].

### 2.8. PET/CT for Prognostication

In view of the close relationship between SSTR expression and treatment options (with non-radioactive and radioactive SSTR analogues), lesions presenting high uptake of [^68^Ga]Ga-DOTA peptides are associated with better outcomes [43,46,56].

On the other hand, low SUVmax values are associated with shorter PFS and OS (overall survival) in patients NETs [57]. Moreover, high overall tumor burden (classified as limited: <five lesions at one site; moderate: >five lesions at two sites; or extensive: >two sites) and tumor heterogeneity are associated with worse prognosis [58].

Pancreatic NENs also show differences in prognosis depending on disease distribution, with worse prognosis if > 25% of the liver and bone metastases are present. In particular, liver metastases at diagnosis significantly decrease survival in most GEP NETs [59].

Visual assessment of SSTR heterogeneity was demonstrated to be both predictive and prognostic in G1 or G2 NET patients (*n* = 65) undergoing PRRT (2–5 cycles). Patients with heterogeneous SSTR expression on target lesions (*n* = 28/65; 44.4%) had a significantly lower OS (*p* = 0.01) and time to progression (TTP; 26 months vs. 54 months, log-rank *p* = 0.013). Moreover, the quality of SSTRs was found to be the only independent prognostic factor for OS (*p* = 0.04; HR = 3.68) and TTP (*p* = 0.03; HR = 3.09) at multivariate analyses [60].

Currently, heterogeneity in SSTR distribution at the lesional level is followed by [^18^F]FDG PET/CT imaging. [^18^F]FDG is a marker of the glycolytic metabolism, and is highly taken up by high-grade, poorly differentiated tumors, such as high-grade G2 (Ki-67 > 10%), NET G3, or NEC. Therefore, [^18^F]FDG-positivity is correlated with the worst prognosis [61,62,63,64,65].

Current EANM/ENETS guidelines [6,8,14] recommend [^18^F]FDG PET/CT in NETs for staging G3 and for higher grade G2 (Ki-67 > 10%). Moreover, [^18^F]FDG PET/CT is recommended in the event of mismatched lesions (dCT-positive/SSTR PET-negative) or rapid progression [18,19].

[^18^F]FDG uptake was reported in around 40% of G1 NETs and nearly all G3 NETs. In thoracic/GEP NETs, the per-patient pooled sensitivity, derived in a recent meta-analysis by Liu et al., was 70% (by grade: 38% G1; 55% G2; 71% G3) [37]. It is interesting to note that most papers investigating the role of [^18^F]FDG PET/CT in NENs often fail to report the timeframe between the first pathological grade assessment and [^18^F]FDG-positivity; therefore, it is difficult to determine whether the [^18^F]FDG uptake was assessed in a true G1 lesion or in an initial dedifferentiation of a previously reported G1 lesion [37].

Optimal diagnostic performances were demonstrated in small-cell lung cancer (SCLC) and Merkel-cell carcinoma (MCC). In particular, in SCLC, a pooled sensitivity and specificity of 97.5 and 98% were reported, respectively, for the assessment of extensive disease pre-therapy [66], whereas in MCC a pooled sensitivity and specificity of 90 and 98%, respectively, were documented [67].

[^18^F]FDG-negativity pre-PRRT in thoracic/GEP NETs was associated with prolonged PFS and OS [68]. On the other hand, higher [^18^F]FDG uptake was associated with worse EFS and OS [69]. Furthermore, the association between higher values of [^18^F]FDG quantitative parameters (such as [^18^F]FDG SUVmax of the primary lesion, metabolic tumor volume, total lesion glycolysis) and inferior EFS, PFS, and OS was demonstrated [70].

In recent years, Chan et al. proposed a grading system where the findings from the combined reading of SSTR-PET/CT and [^18^F]FDG PET/CT are integrated into a single parameter—the “NETPET” score [71]. Despite needing validation in larger prospective studies, this represents a promising biomarker for a wide range of NENs, already showing successful results in bronchial NENs as well as GEP NENs [72].

**Figure 1:**
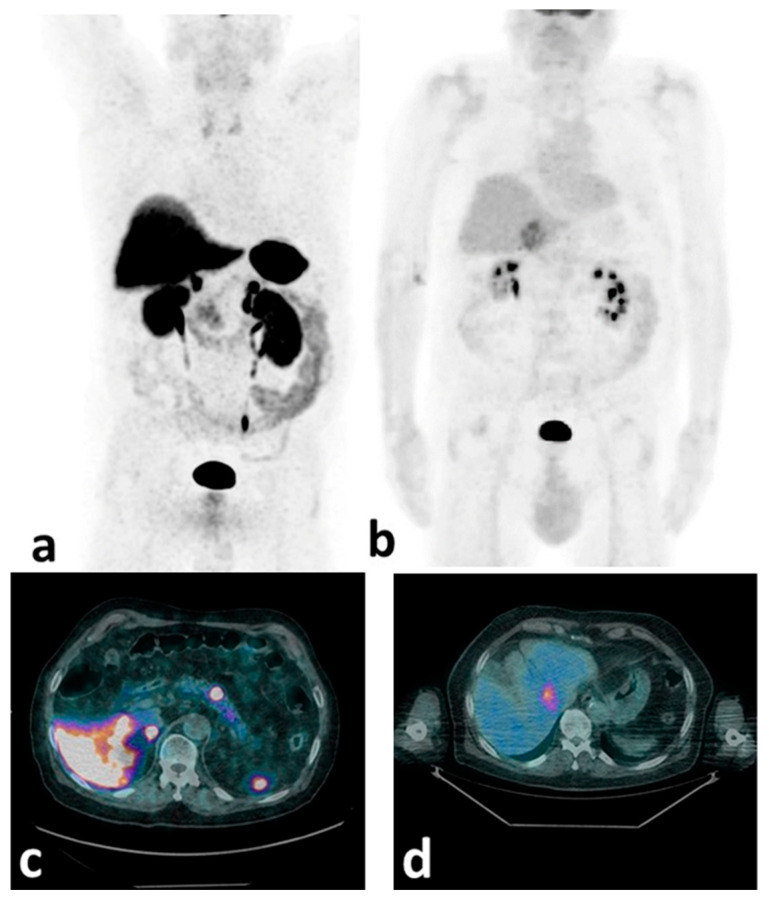
[^68^Ga]Ga-DOTA-NOC and [^18^F]FDG mip (**a**,**b**) and transaxially fused (**c**,**d**) PET/CT images of a patient with a pancreatic G2 NET (Ki-67 = 12%). High SSTR expression is observed in the primary pancreatic body lesion (**a**,**c**), while significant [^18^F]FDG uptake (**b**,**d**) is detected at the liver metastatic level (SSTR-negative). This case shows the potential of the double-tracer PET/CT approach in demonstrating NET de-differentiation.

It is likely that the optimal biological characterization would be achieved with a combined use of SSTR and [^18^F]FDG PET/CT to non-invasively assess disease heterogeneity and predict PRRT response [71,73]. In line with this approach, the ESMO 2020 guidelines [74] also envision the use of double-tracer ([^18^F]FDG +SSTR) PET/CT for all G2–G3 NETs in order to achieve the optimal disease characterization (Figure 1).

In fact, this diagnostic synergy is expected to enable the better selection of the most appropriate treatment option (i.e., PRRT alone vs. PRRT combined with targeted external radiotherapy/chemotherapy/alternative treatments), while also identifying the ratio of differentiated to de-differentiated disease burden at which PRRT fails to be beneficial.

### 2.9. Agonists Labelled with Fluorine

^18^F-labelled SSTR agonists may overcome the practical, regulatory, and economic barriers related to ^68^Ge/^68^Ga generators (e.g., limited availability of FDA- and European Medical Agency (EMA)-approved generators, relatively short ^68^Ga half-life, low activity amounts after single elution that hamper the clinical availability of ^68^Ga-labeled PET tracers). Moreover, fluorinated radiopharmaceuticals could be produced centrally and peripherally distributed; ^18^F has a longer half-life (110 min vs. 68 min of ^68^Ga) and shorter positron range (0.6 mm vs. 3.5 mm of ^68^Ga) suitable for high-resolution imaging [75].

Recently, two radiopharmaceuticals were studied: [^18^F]-AlF-NOTA-octreotide and [^18^F]-SiFAlin-TATE, both presenting biodistribution similar to that of [^68^Ga]-SSTR and preferential uptake in well-differentiated NETs [76,77]. Interestingly, the preferential [^18^F]-AlF-NOTA-octreotide uptake time was 120 min post-injection (p.i.) [76], although it was also acquired at 60 min [78].

A first-in-human study evaluated [^64^Cu]Cu-SARTATE (MeCOSar-Tyr3-octreotate) for imaging 10 NET patients; optimal images were acquired at 4 h p.i., although in 9/10 patients if acquired at 1 h p.i. it was comparable with [^68^Ga]Ga-DOTA-TATE PET/CT [79].

### 2.10. Future Imaging Prospects

#### 2.10.1. Artificial Intelligence

Artificial intelligence could be used to extract image features from medical images in order to build diagnostic, predictive, and prognostic models. In particular, it could be employed to provide a signature (that is, a combination of features) that is not necessarily visible to the naked eye—even an expertly trained one. Although radiomics has shown promising preliminary results in identifying tumor subtypes and aggressiveness, as well as in predicting responses to therapy and the outcomes for patients with various cancers, most of these results have been obtained in small, retrospective, monocentric cohorts, often employing different methods for lesion segmentation and feature extraction [80]. It is currently difficult to generalize the results obtained in published papers due to excessive data heterogeneity; indeed, the application of AI in clinical practice requires absolute methodological harmonization [81]. Thus far, few papers have addressed the use of PET/CT-derived features for the assessment of NENs (Table 2) [82,83,84,85,86,87,88].

#### 2.10.2. SSTR Antagonists

Promising preclinical data support the development of new radiopharmaceuticals that act as antagonists of the SSTR. In vitro studies have demonstrated that antagonists recognize a larger number of binding sites and lack internalization after binding [89]. In humans, antagonists show remarkably low background (especially in SSTR-2-positive organs), resulting in easier lesion detection—especially at the liver level [90,91,92]

Thus far, two theranostic pairs of JR11 have been studied—[^68^Ga]Ga-DOTA-JR11/[^177^Lu]Lu-DOTA-JR11 and [^68^Ga]Ga-NODAGA-JR11/[^177^Lu]Lu-DOTA-JR11—in limited patient cohorts [93,94].

The most promising radiopharmaceutical seems to be [^68^Ga]Ga-NODAGA-JR11 (as compared to [^68^Ga]Ga-DOTA-JR11), showing an SSTR-2 affinity (IC_50_ = 1.2 nmol/L) similar to that of [^177^Lu]Lu-DOTA-JR11 (IC50 = 0.73 nmol/L) [92].

Another promising theranostic pair is [^68^Ga]Ga-DOTA/NODAGA-LM3 and [^177^Lu]Lu-DOTA-LM3, showing high tumor retention and favorable dosimetry [95,96].

## 3. Somatostatin Analogues for Targeted Therapy

### 3.1. PRRT: From Early Studies to Approval

Radiolabeled somatostatin analogues were first employed to diagnose NEN, but they can also be used for treatment when labelled with isotopes emitting cytotoxic radiation. In particular, while [^68^Ga]Ga-DOTA-TOC and [^68^Ga]Ga-DOTA-TATE can be used to image SSTR-expressing NENs, [^90^Y]Y-DOTA-TOC and [^177^Lu]Lu-DOTA-TATE have also been employed for PRRT. Theranostics is an emerging field of nuclear medicine that combines diagnostics with targeted radionuclide therapy, and can be considered to be the epitome of personalized medicine [97]. In fact, the use of PRRT in NEN patients is not new, since it has been used for the treatment of metastatic and inoperable NETs for ~30 years in the setting of clinical trials. This consists of the systemic administration of a stable somatostatin analogue labeled with a β-minus (β-)-emitting radioisotope (e.g., [^90^Y] or [^177^Lu]) that binds to SSTRs overexpressed in target tumor cells [98]. The receptor–peptide complex is internalized by endocytosis, enabling the delivery of cytotoxic radiation directly to the target cells [99,100]. The radiopeptide is retained in the receptor-expressing tumor cells and, due to its relatively low molecular weight, it is rapidly cleared from the blood [101].

The interest in PRRT has steadily increased over the years. An analysis of the literature published from 2000 to 2019 reported that the number of papers on PRRT was more than 20 in 2010, was more than 60 in 2015, and was even higher (111) in 2019 [102].

Early therapeutic studies used [111In]In-DTPA-octreotide in the mid-to-late 1990s [103,104,105]. The idea of Krenning et al. was to use the specific physical characteristics of the Auger emission and conversion electrons of [^111^In] for therapy; the first patient, with an inoperable, metastasized NET (glucagonoma), was successfully treated with high doses of [^111^In]In-pentetreotide [106]. Although there was evidence of some efficacy and minimal toxicity, this treatment presented several disadvantages—it was expensive, it was difficult to deliver a sufficient radioactive dose to the tumor and, due to the ^111^In emission’s short tissue range, it resulted in modest tumor shrinkage. For the abovementioned reasons, ^111^In was soon replaced by novel, more promising alternatives, such as the beta minus emitters yttrium-90 (^90^Y) and lutetium-177 (^177^Lu)—the currently most widely used isotopes combined with DOTA-TOC or DOTA-TATE, respectively.

^90^Y has a half-life of 2.7 days, an average β-energy of 935 keV, and a mean particle penetration range of 3.9 mm. This radionuclide is a long-range pure particle emitter, and may be advantageous in comparison to ^177^Lu when dealing with larger tumors.

^177^Lu is a medium-energy β-emitter with a maximum energy of 0.5 MeV and a maximal tissue penetration of 2 mm; ^177^Lu also emits low-energy γ-rays at 208 and 113 keV, with 10 and 6% abundance, respectively. Early-phase PRRT trials employed either [^90^Y]Y-DOTA-TOC or [^177^Lu]Lu-DOTA-TATE, obtaining good response rates—especially in patients with GEP NETs (disease control rates ranging from 66% to 92%) [107,108,109,110,111,112,113,114,115,116,117].

To improve PRRT efficacy, the combination of ^90^Y and ^177^Lu treatment regimens has been proposed, in order to exploit the different soft tissue penetration range of the β-emitted radiation [118].

In addition to efficacy studies, many early papers investigated the toxicity profile of PRRT, especially focused on the effects on the kidneys and bone marrow. Many papers reported the long-term incidence of bone marrow toxicity following [^177^Lu]Lu-DOTA-TATE and [^90^Y]Y-DOTA-TOC PRRT; however, hematological toxicity—such as acute leukemia or myelodysplastic syndrome (AL or MDS)—was reported in a minority of patients. In a study including 1109 patients, severe toxicity was observed in 12.8% of patients (3–4 hematological toxicity: 0.1% of AL and 0.1% of MDS) [119]. Overall, the risk of the development of myeloproliferative disorders was reported to be 2% [119,120,121,122].

Due to the mainly renal excretion of these tracers, renal toxicity was also reported, even when a kidney protection protocol (consisting of the infusion of positively charged aminoacidic infusion) was implemented. This latter strategy, however, reducing the radiopharmaceutical’s renal uptake, was reported to reduce the frequency of severe renal toxicity (grade 3/4) to less than 3% of cases [123,124,125].

Unfortunately, early studies were mainly retrospective phase I-II trials, and were difficult to compare due to their heterogeneous patients cohorts and treatment schemes (in terms of total injected activity, activity per cycle, time intervals between cycles, and number of cycles) [109,110,112,126,127,128,129]. In 2017, the publication of the NETTER-1 trial results significantly changed the clinical employment of PRRT, and paved the way for the approval of [^177^Lu]Lu-DOTA-TATE PRRT in the United States and Europe [130]. The NETTER-1 study was a multinational, open-label, phase III randomized clinical trial that evaluated PRRT safety and efficacy in patients with advanced, progressive, SSTR-positive, well-differentiated midgut NETs. A total of 229 patients were randomized a the PRRT arm (treated with four infusions of 7.4 GBq [^177^Lu]Lu-DOTA-TATE every 8 weeks plus 30 mg of octreotide every 4 weeks for symptom control) and a control arm (treated with off-label FDA-approved high-dose (60 mg) octreotide every 4 weeks). Treatment with [^177^Lu]Lu-DOTA-TATE was associated with a 79% reduction in the risk of progression or death compared to the control arm. PFS was significantly higher in the PRRT arm, and the interim analysis even suggested longer OS; furthermore, overall toxicity was limited [131]. Although this study included only patients with midgut NETs, the subsequent approval of [^177^Lu]Lu-DOTA-TATE was also extended to pancreatic NETs following the publication of the Erasmus study, reporting the safety and efficacy of [^177^Lu]Lu-DOTA-TATE in a large population of patients (*n* = 610). Among the subgroup of 443 patients analyzed for efficacy and survival, patients with primary pancreatic NETs (*n* = 133) had the longest OS (71 months) [132].

[^177^Lu]Lu-DOTA-TATE is now approved [130] for the treatment of unresectable or metastatic, progressive, well-differentiated (G1 and G2), SSTR-positive GEP NETs in adults in Europe and the United States, and for bronchial NENs in the United States. Recently, the NETTER-1 study’s final findings (at 5 years after the last patient was randomized) were presented during the 2021 American Society of Clinical Oncology (ASCO) annual meeting. [^177^Lu]Lu-DOTA-TATE treatment was associated with a clinically and statistically significant improvement in PFS (HR: 0.18, *p* < 0.0001). Interestingly, although longer OS was observed in the treated arm, there was no statistically significant difference in the median OS between the [^177^Lu]Lu-DOTA-TATE arm (48.0 months; 95% CI: 37.4–55.2) and the control arm (36.3 months, 95%CI: 25.9–51.7). This can likely be explained by the high rate (36%) of crossover of progressing patients from the control arm to the PRRT arm. No new safety signals emerged during the 5-year long-term follow-up (in particular, no new cases of MDS or acute leukemia were reported in the long-term follow-up) [131].

Collecting the evidence gathered so far, PRRT should be considered as a systemic, effective, and safe treatment option, and has been included in the therapeutic algorithms of the main scientific societies. However, there are still many issues to be clarified regarding the most efficient positioning of PRRT in the therapeutic algorithm sequence, the eligibility criteria for PRRT, treatment scheduling, dosimetry estimation, and response assessment criteria.

### 3.2. PRRT: Impact on Quality of Life

NET patients’ symptoms can be related to tumor growth as well as hormone production. The carcinoid syndrome (CS) is characterized by skin flushing, diarrhea, abdominal pain, fast heart rate, and bronchospasm. Especially in severe CS, symptomatic control can be challenging; thus, quality of life (QoL) may be significantly affected by symptoms related to tumor growth, as well as by hormone production [133]. Somatostatin analogues are typically used for the control of tumor growth and hormonal symptoms [134]. To date, the effectiveness of PRRT in the treatment of advanced GEP NETs in terms of controlling the CS and improving QoL is supported by various published trials, as well as by clinical experience [131,135,136,137,138,139,140]. In a study that evaluated the QoL of 265 patients, the authors found that global health status (GHS), QoL, Karnofsky performance score (KPS), and symptoms significantly improved after PRRT; moreover, there was no significant decrease in QoL in patients who had no symptoms before therapy. In patients who had suboptimal scores for GHS/QOL or symptoms before therapy, a clinically significant improvement was reported [135]. Moreover, in an analysis from the NETTER-1 phase III study, the impact of [^177^Lu]Lu-DOTA-TATE treatment on time to deterioration (TTD) in health-related QoL was assessed; in addition to improving PFS, [^177^Lu]Lu-DOTA-TATE provided a significant QoL benefit for patients with progressive midgut NETs as compared to high-dose octreotide LAR [136]. In particular, the authors reported statistically significant reductions from baseline in the mean number of days with all three core symptoms of patients with progressive midgut NETs (i.e., abdominal pain, diarrhea, and flushing), as compared with high-dose octreotide LAR treatment. TTD was significantly longer in the [^177^Lu]Lu-DOTA-TATE arm versus the control arm (for GHS, physical functioning, role functioning, fatigue, pain, diarrhea, disease-related worries, and body image). Differences in median TTD were clinically significant in several domains: 28.8 months versus 6.1 months for GHS, and 25.2 months versus 11.5 months for physical functioning [136]. In a more limited cohort of 22 patients with refractory CS syndrome and radiologically stable or newly diagnosed disease, PRRT effectively reduced diarrhea and flushing. The authors concluded that PRRT should be considered for symptomatic treatment of CS insufficiently controlled with somatostatin analogues [141].

### 3.3. PRRT: Eligibility and Contraindications

The first guidelines on the clinical use of PRRT were published in 2013 thanks to a joint effort of the EANM, International Atomic Energy Agency, and Society of Nuclear Medicine and Molecular Imaging, followed by the ENETS Consensus Guidelines (2017) and the North American Neuroendocrine Tumor Society (NANETS) Consensus Statement on Patient Selection for PRRT (2020) [8,142].

All guidelines agree on the need for multidisciplinary discussion of each case in order to evaluate PRRT eligibility. Inoperable/metastatic well-differentiated G1–G2 NETs are the best candidate patients. Currently, PRRT is offered as a second-line treatment, after failure of first-line treatment with somatostatin analogues, in patients with a KPS > 50 and expected survival > 3 months. In all cases, significant SSTR expression should be demonstrated by SSTR imaging (the tumor uptake should be at least as high as normal liver uptake: Krenning score ≥ 2) [49].

As a result of the publication of the NETTER-1 phase III study, PRRT with [^177^Lu]Lu-DOTA-TATE is currently approved for adult patients with unresectable or metastatic, progressive, well-differentiated (G1 and G2), SSTR-positive GEP NETs [131]. Currently, GEP NET patients can be treated with PRRT as a second-line treatment, after the failure of first-line somatostatin analogues.

PRRT’s absolute contraindications include pregnancy, hypersensitivity to the active substance or to any of the excipients contained in the radiopharmaceutical, severe acute concomitant illnesses (e.g., severe cardiac or liver impairment), and severe unmanageable psychiatric disorders. Breastfeeding should be discontinued before PRRT. Severely compromised renal function represents a relative contraindication [98]; the ENETS recommends that patients should have a creatinine clearance of ≥50 mL/min [8], while the NANETS suggests that a creatinine clearance of ≥30 mL/min is sufficient [142]. As mentioned above, aminoacidic infusion is recommended because it notably reduces the radiopharmaceutical’s renal uptake; renal toxicity with [^177^Lu]Lu-DOTA-TATE is negligible when prophylactic amino acids are used, even in patients with a glomerular filtration rate < 50 mL/min. Moreover, co-infusions of arginine/lysine result in substantially lower rates of nausea/vomiting compared with commercial amino acid formulations designed for parenteral nutrition [143]. Literature data are also in favor of extending both the amino acid solution infusion time and the amino acid protection to over 2 days, in order to further reduce renal toxicity.

PRRT’s absolute contraindications include pregnancy, hypersensitivity to the active substance or to any of the excipients contained in the radiopharmaceutical, severe acute concomitant illnesses (e.g., severe cardiac or liver impairment), and severe unmanageable psychiatric disorders. Breastfeeding should be discontinued before PRRT. Severely compromised renal function represents a relative contraindication [98]; the ENETS recommends that patients should have a creatinine clearance of ≥50 mL/min [8], while the NANETS suggests that a creatinine clearance of ≥30 mL/min is sufficient [142]. As mentioned above, aminoacidic infusion is recommended because it notably reduces the radiopharmaceutical’s renal uptake; renal toxicity with [^177^Lu]Lu-DOTA-TATE is negligible when prophylactic amino acids are used, even in patients with a glomerular filtration rate < 50 mL/min. Moreover, co-infusions of arginine/lysine result in substantially lower rates of nausea/vomiting compared with commercial amino acid formulations designed for parenteral nutrition [143]. Literature data are also in favor of extending both the amino acid solution infusion time and the amino acid protection to over 2 days, in order to further reduce renal toxicity.

Sufficient bone marrow reserves are required (grade 1–2 hematological toxicity is usually accepted). An accurate pre-administration evaluation should be carried out in order to select patients at higher risk of renal or bone marrow toxicity; at least 2–4 weeks and directly before administration of each [^177^LuLu]-DOTA-TATE dose, the liver and kidney function as well as hematological parameters must be checked. When dose-modifying toxicity occurs, either prolongation of the treatment interval (up to 16 weeks) or treatment discontinuation may become necessary.

Among the relative contraindications, the ENETS guidelines indicate the presence of mismatched lesions on [^18^F]FDG and SSTR PET/CT ([^18^F]FDG-positive and [^68^Ga]Ga-DOTA-peptide-negative), as well as cases with low SSTR expression or mesenteric disease.

Although [^18^F]FDG PET/CT is not part of the standard pre-PRRT protocol, it is well known that a lower response to PRRT is expected in patients with matched ([^18^F]FDG- and [^68^Ga]Ga-DOTA-peptide-positive) lesions, representing a contraindication to PRRT alone when lesions are mismatched.

In the recent EANM Focus 3, the use of [^18^F]FDG PET/CT before PRRT was recommended in the event of non-resectable/disseminated G2 or G3 NETs, in order to exclude mismatched lesions, or as a prognostic factor. The possibility of treating patients with G3 NETs has been suggested by several positive experiences [40,50,51]. However, current international guidelines provide different indications; the ENETS guidelines state that PRRT may be considered for G3 NETs (although further data are needed) and even NECs with SSTR-expressing disease [8], while the NANET guidelines emphasize the limited data on PRRT in these subgroups [142]. It is also worth mentioning that current evidence suggests that PRRT may potentially be a superior first-line therapeutic option for G3 NET patients with Ki-67 ≤ 55%, as compared to platinum-based chemotherapy (providing high SSTR expression with no discordant FDG-avid lesions) [144].

Further data are certainly needed in order to determine the real potential benefit of this treatment option in G3 NET patients; the results of the ongoing NETTER-2 study (evaluating PRRT as a first-line treatment in G2 and G3 NETs) will likely help elucidate this issue (https://clinicaltrials.gov/ct2/show/NCT03972488 accessed on 6 November 2021).

Another aspect to consider in the selection of patients is the presence of extensive peritoneal carcinomatosis (PC), for the potential risk of radiation-induced bowel obstruction [142]. This is another area of debate; the NANETS guidelines indicate that patients with bulky mesenteric tumors and PC are potentially at higher risk for PRRT, while the ENETS guidelines do not address this topic. Some literature data suggest that PRRT should be adopted with caution in GEP NETs with diffuse PC. A retrospective, single-center analysis performed in 135 GEP NENs with diffuse PC treated with PRRT showed peritoneal progression in 37.5% of cases, and bowel obstruction or ascites in 28.1% [145]. One study recently reported a bowel obstruction rate of 5% among patients with baseline mesenteric or peritoneal disease [146]. Premedication with low-dose steroids started after PRRT and continued for 2–4 weeks after therapy has been proposed to prevent complications in patients with PC [142,145].

The final indication for PRRT needs to be discussed in a multidisciplinary setting (oncologists, nuclear medicine physicians, radiologists, endocrinologists, surgeons, gastroenterologists, endoscopists, etc.), while also considering the other available treatment options (e.g., defining the most appropriate treatment sequence), as well as various factors that are known to potentially affect PRRT response (e.g., performance status, primary tumor site, previous treatments, biomarkers, grading, tumor burden, [^18^F]FDG and [^68^Ga]Ga-DOTA peptides PET/CT uptake: match/mismatch) [62,64,112,138,147,148,149,150].

### 3.4. Personalized Dose or Fixed-Dose Scheme?

The possibility of personalizing the treatment scheme for each patient would be desirable. From a technical point of view, dosimetry is feasible; the main focus of dosimetry is to improve efficacy by sparing at-risk organs (reducing toxicity), and to optimize the dose delivered to the tumor. Since ^90^Y does not emit suitable γ-rays, imaging after administration is difficult; on the other hand, ^177^Lu emits low-energy γ-rays that allow dosimetric evaluation between cycles.

As mentioned above, the kidneys and the bone marrow represent critical targets of long-term PRRT toxicity. However, it was not possible to identify a definitive threshold for the risk of developing nephrotoxicity or hematotoxicity. In clinical practice, a threshold of 23 Gy, derived from radiation therapy studies, is generally used for kidneys, and a threshold of 2 Gy, adapted from radioiodine therapy, is used for bone marrow. However, there is no clear evidence that these cutoffs are optimal for PRRT [151,152,153,154].

Although the results of the final analysis of the NETTER-1 study indicate that PRRT is safe and effective, and rarely causes serious toxicity [155], there is evidence that dosimetric evaluation can play an important role—especially in patients with risk factors for renal or hematological toxicity, or those presenting with comorbidities [63,120,121]. Moreover, several studies have shown that additional PRRT cycles (salvage therapy setting) or re-treatments can be safely performed following dosimetric estimations [156,157,158,159].

Dosimetric evaluation was previously focused mainly on normal organs (so as to limit side effects), but in recent years it has been used to better understand the correlation between tumors’ absorbed dose and tumor response. For pancreatic neuroendocrine tumors, a significant correlation between tumor dose and tumor shrinkage was reported [160], which was however not confirmed in 25 patients with small intestine NENs [161].

Despite the efforts and the numerous publications to further improve PRRT by using dosimetry [154], a recent survey in European centers applying radionuclide therapies has shown that dosimetry is rarely or never used in more than half of centers; moreover, its results barely influence treatment [162]. One of the reasons is that dosimetry is time-consuming, labor-intensive, expensive, and costs patient time (multiple acquisitions are required).

Nevertheless, each country has already adopted or will have to adopt the forthcoming European Council Directive 2013/59/Euratom that, in article 56, mandates the use of dosimetry-based treatment planning and verification of the absorbed doses delivered, taking into account that doses to non-target volumes and tissues should be as low as reasonably achievable and consistent with the intended radiotherapeutic purpose of the exposure. In view of the Council Directive (2013/59/Euratom), there is a need to increase the possibilities and benefits of a higher degree of implementation of dosimetry, and this implies having more practical, robust, and standardized dosimetry methods for wider clinical application.

### 3.5. New Trends and Future Perspectives of PRRT for the Treatment of NETs

A higher risk of disease progression on PRRT has been observed in NET patients with lesions ≥ 3 cm in size or that show [^18^F]FDG positivity [64,163], suggesting the need to improve PRRT’s debulking capability as well as its efficacy in some patient categories [164]. Ongoing studies are exploring different strategies to improve outcomes on PRRT of patients with NENs, such as using different peptides, radionuclides, and carriers, by combining PRRT with radiosensitizers, or by optimizing the treatment sequence.

#### 3.5.1. Improving PRRT: Investigational Peptides, Radionuclides, and Carriers

[^177^Lu]Lu-edotreotide (DOTA-TOC) is an investigational radiolabeled somatostatin analog that showed a favorable safety profile and promising efficacy in a retrospective study, obtaining a median PFS of 34.5 months [165,166]. The COMPETE study is an ongoing multicenter randomized controlled phase III trial of [^177^Lu]Lu-edotreotide versus everolimus in patients with progressing advanced GEP NETs (expected *n* = 300), with PFS as the primary endpoint (NCT03049189).

As mentioned above, SSTR antagonists may offer advantages over currently employed agonists [89,93]. The safety of satoreotide tetraxetan (formerly known as [^177^Lu]Lu-DOTA-JR11)—an SSTR antagonist—was investigated in a phase I clinical trial in 40 patients with well-differentiated NETs, who received two cycles of the investigational agent 3 months apart (NCT02609737) [167]. After 20 patients were enrolled (with 6 patients receiving one cycle and 14 receiving two cycles), grade 4 myelosuppression was observed in 57.1% of cases after cycle two, so that a protocol amendment was needed to limit the bone marrow dose to 1 Gy and reduce the dose of cycle 2 by 50%. Preliminary efficacy data reported an ORR of 45% and a median PFS of 21.0 months [167].

Because of their higher linear energy transfer, α-emitting radionuclides are an attractive agent to improve PRRT efficacy in NETs, despite the potential risk of the release of daughter radionuclides from the chelator, which can have a long half-life and, thus, result in increased rates of severe toxicity. Actinium-225 [^225^Ac], bismuth-213 ([^213^Bi]), and lead-212 ([^212^Pb]) are three experimental α-emitters that have been shown to be active in patients with NENs refractory to ^90^Y- or ^177^Lu-based PRRT [168,169,170]. In particular, a phase I trial of [^212^Pb]Pb-DOTA-TATE in treatment-naïve patients with NETs is ongoing (NCT03466216) [170].

The development of novel carriers could improve PRRT efficacy by optimizing pharmacokinetics and dosimetry. A phase I study of [^177^Lu]Lu-DOTA-EB-TATE in patients with NETs reported increased circulation time and tumor uptake compared to [^177^Lu]Lu-DOTA-TATE, but also increased kidney and bone marrow dose delivery (NCT03478358) [171].

#### 3.5.2. Combining PRRT: Chemotherapy and Other Radiosensitizers

To increase the damage caused by radiation to tumor cells and increase their killing, different radiosensitizers can be administered concomitantly to PRRT. One way to achieve this aim—and the most extensively explored way—is to combine PRRT with chemotherapy, such as a fluoropyrimidine, namely, 5-fluorourcil (5FU) or capecitabine (CAP) and/or temozolomide (TEM) [164,172]. Peptide receptor chemo-radionuclide therapy (PRCRT) in patients with well-differentiated GEP NETs treated within early-phase clinical trials provided ORRs as high as 24–53% [173,174,175], which also exceeded 80% in the subgroup of patients with pancreatic NETs receiving PRCRT with TEM—likely because of the role of MGMT (O(6)-methylguanine-DNA methyltransferase) promoter methylation in these patients [176]. In these studies, PRCRT was safe and its toxicity was manageable, with mainly grade 2 and grade 3 subacute hematological toxicity and nausea. The CONTROL NETS study is an ongoing randomized non-comparative phase II trial of the combination of CAPTEM and [^177^Lu]Lu-DOTA-TATE, or either treatment alone, in patients with well-differentiated G1–2 pancreatic and midgut NETs (NCT02358356) [177]. Preliminary data on small bowel NET (*n* = 47) patients showed a 15-month PFS rate—the primary endpoint—of 90% and 92% and an ORR of 31% vs. 15% in the PRCRT arm (*n* = 33) and in the PRRT arm (*n* = 14), respectively. In the cohort of patients with pancreatic NETs (*n* = 28), the 12-month PFS rate—the primary endpoint—was 76% and 67% with an ORR of 68% vs. 33% in the PRCRT arm (*n* = 19) and in the CAPTEM arm (*n* = 9), respectively. Despite similar efficacy in terms of PFS, PRCRT yielded numerically higher ORR alongside greater toxicity, mainly affecting the bone marrow. PRCRT can also be a useful strategy in the specific setting of patients with [^18^F]FDG-positive NENs, as there are data showing that PRCRT with 5FU is feasible, active (with an ORR of 30%), and tolerable, as grade 3–4 adverse events were observed in approximately 8% of the 52 patients with [^18^F]FDG-avid NENs of mostly GEP origin included in a retrospective analysis [178].

The efficacy of PRRT in patients with well-differentiated NETs with a high proliferation index (G2 NET with Ki-67 > 10% and G3 NET) is still unclear. To clarify the role of PRRT in this setting, the NETTER-2 study—a randomized open-label multicenter phase III trial of [^177^Lu]Lu-DOTA-TATE compared to high-dose octreotide LAR in patients with G2 and G3 advanced GEP NETs—is ongoing (NCT03972488).

The COMPOSE study—a prospective randomized controlled open-label multicenter phase III trial of [^177^Lu]Lu-edotreotide compared to standard of care (including everolimus and chemotherapy) in the same population—has a similar objective (NCT04919226).

Some retrospective series have reported the use of PRCRT (with 5FU, CAP, or CAPTEM) in cohorts that also included patients with G3 NENs, with an ORR of 30–47% [137,179,180]. Nevertheless, the rate of patients presenting disease progression as the best response was high, ranging from 20% to 26%, suggesting either a need for careful patient selection, or that the intrinsic aggressiveness of G3 NETs makes PRRT or PRCRT unsuitable for these patients.

Poly(ADP-ribose) polymerase inhibitors (PARPis) are widely used, alone or in combination, in different tumor types, because they inhibit homologous recombination—a pathway of the DNA damage response repair machinery—and result in cell death under certain conditions [181]. The rationale of using PARPis as radiosensitizers lies in the fact that tumor cells are not able to repair the DNA single-strand break induced by [^177^Lu]Lu-DOTA-TATE in the presence of PARPis, resulting in DNA double-strand break and cell death, as also shown in preclinical NET models [182]. Olaparib, a PARPi, will be administered twice daily from 2 days prior to the start to 4 weeks after the end of [^177^Lu]Lu-DOTA-TATE treatment in patients with progressive GEP NETs who have not previously received PRRT in a phase I/II trial (NCT04086485). Similarly, radionuclide reductase is a rate-limiting enzyme in DNA synthesis and repair, and its inhibition might sensitize NET cells to radiation damage [183]. A phase I study of [^177^Lu]Lu-DOTA-TATE and triapine—a radionuclide reductase inhibitor—in PRRT-naïve patients with well-differentiated GEP NETs is ongoing [184].

#### 3.5.3. The Place Where PRRT Belongs: Concepts of Sequencing

There is no current consensus on the sequencing of approved therapies, particularly with respect to PRRT. Available evidence is mostly derived from retrospective and, therefore, potentially biased series [8,74,185]. A recent retrospective multicenter Italian study showed that [^177^Lu]Lu-DOTA-TATE, as well as non-conventional-dose somatostatin analogues [186], are better tolerated than chemotherapy or everolimus, with no significant difference in PFS [187]. Because a recent network meta-analysis showed that [^177^Lu]Lu-DOTA-TATE had the highest probability of being associated with the longest PFS as compared to other approved treatments [188], it is not surprising that ongoing studies—namely, the NETTER-2 (NCT03972488) and COMPETE trials (NCT03049189)—aim at first-line positioning of PRRT. Nevertheless, shifting PRRT to earlier in the treatment algorithm for patients with NETs is associated with poorer tolerance of subsequent treatments, such as everolimus [189], or might not be associated with improved outcomes, e.g., in patients with pancreatic NETs [190]. As surgery is also acknowledged to be the only potentially curative treatment for NET patients in the metastatic setting, some patients with marginally unresectable NETs can benefit from neoadjuvant treatment and be considered as surgical candidates. PRRT’s role in the neoadjuvant setting has been reported in a retrospective study, with the interesting collateral finding of a reduced incidence of subsequent liver metastasis. To confirm these findings, a prospective phase II trial of neoadjuvant PRRT with [^177^Lu]Lu-DOTA-TATE followed by surgery for patients with resectable pancreatic NETs is currently ongoing (NCT04385992). The feasibility of PRRT re-treatment presents an appealing opportunity in NET patients who initially respond and then experience disease progression, given the long-term benefit in terms of PFS and the good subjective patient tolerance. PRRT re-treatment is not currently recommended by guidelines, but there are some retrospective reports about its safety and feasibility, potentially leading to PFS of 14–22 months [156,191].

## 4. Conclusions

NENs are rare and heterogeneous cancers that can be managed using the theranostic principle. From the diagnostic perspective, SSTR PET/CT is the gold standard modality for imaging of NETs (i.e., identification of primary lesion, disease extension, selection for PRRT). Intra-patient and interlesional SSTR heterogeneity (e.g., metastases may differ from the primary tumor; each metastasis may derive from a different clone, with variable SSTR expression) may require additional [^18^F]FDG PET/CT to assess the glycolytic metabolism, impacting both management and prognosis. On the therapeutic side, PRRT should be considered as a systemic, effective, and safe treatment option, and is included in the therapeutic algorithms of the main scientific societies. The final indication for PRRT needs to be discussed at a multidisciplinary level, while also considering the other available treatment options as well as various factors that are known to potentially reduce its efficacy (e.g., performance status, primary tumor site, previous treatments, biomarkers, grading, tumor burden, and [^18^F]FDG and [^68^Ga]Ga-DOTA peptides PET/CT uptake: match/mismatch). Ongoing studies are focused on the optimal treatment sequence, a better definition of PRRT selection criteria, strategies to reduce treatment-induced toxicity, and novel radiopharmaceuticals (e.g., fluorinated analogues, antagonists).

## Figures and Tables

**Table 1:** Diagnostic performance of meta-analysis data on 68Ga-DOTA peptides PET.

NENs	References	Relevant Study Details	Per-Patient				Per-Lesion	Comments	Number of Included Studies	Number of Included Pts
			Pooled Sens% (95% CI)	Pooled Spec% (95% CI)	DR (%) or ROC-AUC		DR or Sens or Spec (%) or ROC-AUC			
**SUSPECTED NET**	Sing 2018 [29]	Initial diagnosis	91 (85–94)	94 (86–98)					22	na
	Sing 2018 [29]	Staging and restaging	78.3 to 100	83 to 100						
**THORACIC and/or GEP NET**	Treglia, 2012 [30]	All tracers	93 (91–95)	91 (82–97)	-AUC 0.96				16	567
	Geijer 2013 [31]	All tracers	93 (91–94)	96 (95–98)	-AUC 0.98 (0.95–1.0)				22	2105
	Yang 2014 [32]	TOC	93 (89–96)	85 (74–93)	-AUC 0.96				10	416
		TATE	96 (91–99)	100 (82–100)	AUC 0.98					
	Deppen, 2016 [33]	TATE vs. octreotide and conventional imaging	90.9 (81.4–96.4)	90.6 (77.8–96.1)				TATE superiority	42	
	Bauckneht 2020 [34]	Primary pancreatic NET, all tracers	79.6 (71–87)	95 (75–100)	DR 81 (65–90)		DR 92 (80–97)		18	1143
	Piccardo 2021 [35]	Intestinal NET (head to head, all tracers vs. [^18^F]FDOPA)	88	na			Sens 82	[^18^F-]F-DOPA superiority only per lesion (95%) but not per patient (83%)	6	112
	Jiang 2019 [36]	Pulmonary carcinoids (all tracers vs. [^18^F]FDG)	90 (82.0–95.0)					Higher SSTR uptake in typical carcinoids than atypical ones; FDG overall sens 71 (66.0–76.0)	14	352
	Liu, Hell 2020 [37]	All grades, all tracers	92 (0.89–0.95)	91 (0.83–0.95)	AUC 0.96 (0.94–0.98)		Sens 95 (0.86–0.98); spec93 (0.83–0.97); AUC 0.98 (0.96–0.99)		30	3401
		G1	92							
		G2	90							
		G3	58							
		TOC	95	91						
		TATE	92	88						
		NOC	87	90						
**CUP-NET**	Ma 2021 [38]	All tracers	82	55	AUC 69; DR 61;				10	484
	De Dosso 2019 [39]	All tracers			DR 56 (48–63)			Most frequent sites of CUP: bowel and pancreas.	12	383
**PGL**	Kan 2018 [40]	All tracers vs. FDG without germline mutations;	95 (0.92–0.97)	87 (0.63–0.96)	AUC 0.78 (0.74–0.81)			FDG: sens 85 and spec 55; AUC 0.88 (0.85–0.91)	17	629
		with germline mutations	97	79						
	Han 2019 [41]	All tracers vs. DOPA, FDG, MIBG			DR 93 (91–95)			DR [^18^F]FDOPA PET 80 (69–88), [^18^F]FDGPET 74 (46–91), and [^123^/^131^I]MIBG scan 38 (20–59). SSTR DR superiority, particularly head and neck PGL.	9	215
**rMTC**	Treglia 2017 [42]	All tracers					DR 63.5 (49–77)	SSTR PET DR 83% if calcitonin > 500 ng/L; [^18^F]FDOPA is recommended in this setting	9	

Table legend: NEN: neuroendocrine neoplasia; NET: neuroendocrine tumor; GEP: gastroenteropancreatic; CUP: cancer of unknown primary site; PGL: paraganglioma; rMTC: recurrent medullary thyroid cancer; CI: confidence interval; DR: detection rate; sens: sensitivity; spec: specificity; SSTR: somatostatin receptor; ROC: receiver operating characteristics; AUC: area under the curve; na = not available.

**Table 2:** PET/CT-derived radiomic features for the assessment of NENs.

Author	Journal, Publication Date	Ref	Clinical Setting	Primary Site	Modality	Tracer	Number of Patients	Methods
Atkinson C	*Frontiers in Oncology*, 2021	[82]	Prognosis/PRRT	Multiple *	PET/CT	[^68^Ga]Ga-DOTA-TATE	44	TexRAD research software, Cambridge, UK
Bevilacqua A	*Diagnostics* (Basel), 2021	[87]	Tumor grade prediction	pNET	PET/CT	[^68^Ga]Ga-DOTA-NOC	58	ImageJ + in-house-generated model
Thuillier P	*Biomedicines*, 2021	[83]	Pathological classification	Lung NEN	PET/CT	[^18^F]FDG	44	LIFEx v.6.0 (IMIV/CEA, Orsay, France
Liberini V	*EJNMMI Physics*, 2021	[84]	Segmentation	Multiple $	PET/CT	[^68^Ga]Ga-DOTATOC	49	LifeX v.4.81 (IMIV/CEA, Orsay, France
Liberini V	*Frontiers in Medicine* (Lausanne), 2021	[85]	PRRT outcome	G3 NET (pancreas, rectum)	PET/CT	[^68^Ga]Ga-DOTATOC/[^18^F]FDG	2	LIFEx v.5.10 (IMIV/CEA, Orsay, France)
Mapelli P	*Nuclear Medicine Communications*, 2020	[88]	Pre-operative risk assessment	pNEN	PET/CT	[^68^Ga]Ga-DOTATOC/[^18^F]FDG	61	Chang-Gung Image Texture Analysis software package
Weber M	*BMC Cancer*, 2020	[86]	Treatment-related changes		PET/MRI	[^68^Ga]Ga-DOTATOC	18	LIFEx v.6.0 (IMIV/CEA, Orsay, France

Legend: * = carcinoid, pancreatic, thyroid, head and neck, catecholamine-secreting, and unknown primary NET; $ = GEP + lung + other; Ref = reference.
